# Construction of miRNA-regulated drug-pathway network to screen drug repurposing candidates for multiple sclerosis

**DOI:** 10.1097/MD.0000000000029107

**Published:** 2022-03-18

**Authors:** Xiaotong Kong, Jianjian Wang, Yuze Cao, Xiaoyu Lu, Huixue Zhang, XiaoMing Zhang, Chunrui Bo, Ming Bai, Shuang Li, Yang Jiao, Lihua Wang

**Affiliations:** ^a^ *Department of Neurology, The Second Affiliated Hospital, Harbin Medical University, Harbin, Heilongjiang Province, China,* ^b^ *Department of Neurology, Peking Union* *Medical College Hospital, Beijing 100730, China.*

**Keywords:** drug repurposing, gene, miRNA, multiple sclerosis, pathway network

## Abstract

Given the high disability rate of multiple sclerosis (MS), there is a need for safer and more effective therapeutic agents. Existing literature highlights the prominent roles of miRNA in MS pathophysiology. Nevertheless, there are few studies that have explored the usefulness of existing drugs in treating MS through potential miRNA-modulating abilities.

The current investigation identifies genes that may exacerbate the risk of MS due to their respective miRNA associations. These findings were then used to determine potential drug candidates through the construction of miRNA-regulated drug-pathway network through genes. We uncovered a total of 48 MS risk pathways, 133 MS risk miRNAs, and 186 drugs that can affect these pathways. Potential MS risk miRNAs that are also regulated by therapeutic candidates were hsa05215 and hsa05152. We analyzed the properties of the miRNA-regulated drug-pathway network through genes and uncovered a number of novel MS agents by assessing their respective Z-values.

A total of 20 likely drug candidates were identified, including human immunoglobulin, aspirin, alemtuzumab, minocycline, abciximab, alefacept, palivizumab, bevacizumab, efalizumab, tositumomab, minocycline, etanercept, catumaxomab, and sarilumab. Each of these agents were then explored with regards to their likely mechanism of action in treating MS.

The current investigation provides a fresh perspective on MS biological mechanisms as well as likely treatment strategies.

## 1. Introduction

Multiple sclerosis (MS) is an autoimmune inflammatory demyelinating disease of the central nervous system (CNS).^[1]^ The immunological mechanism of MS is characterized by focal lymphocytic infiltration leading to myelinoclasis.^[2]^ MS possesses a complex pathophysiology that involves a combination of genetic susceptibility and genetic polymorphism.^[3]^ A myriad of immune system molecules and cells are implicated in MS.^[4]^ Both adaptive immune system and innate immune system participate in inflammation in MS. When antigen presenting cells recognized the autoantigen in CNS, T and B cells will be activated and play key roles in the pathogenesis of MS.^[3]^ Meanwhile, the activation of microglia and infiltration of lymphocyte and monocyte lead to axonal demyelinating, axonal loss, gliosis, and white matter injury. According to the 2017 McDonald criteria, MS can be divided into the following types: relapsing remission type multiple sclerosis (RRMS), secondary progressive multiple sclerosis (SPMS), primary progressive multiple sclerosis, and others.^[5]^ Given of the high disability rates of MS, safer and more effective medicines for MS are necessary.^[6]^ Current treatment strategies for MS focus on reducing recurrence risk and delaying disability progression. The introduction of disease-modifying drugs that aim to halt MS progression have benefited a significant proportion of patients with this debilitating disease.^[7]^

In recent years, numerous investigations allude to the involvement of miRNAs in immune regulation, especially in the context of autoimmune conditions.^[8]^ There are several investigations supporting that miRNAs play key roles in pathophysiology of MS.^[9,10]^ One study reports the relationship between patient disease severity and miR-326, which was hypothesized to augment TH-17 differentiation through Ets-1 targeting.^[11]^ Additionally, macrophage activation was demonstrated to be modified by miRNA-34a, miRNA-155, and miRNA-326 targeting of the 3′-untranslated region of CD47.^[12]^ In an experimental autoimmune encephalomyelitis (EAE) model, disease severity was alleviated and the rates of CNS inflammation were reduced with miR-155 silencing or anti-miR-155 treatment, a phenomenon that is hypothesized to work by modulating the inflammatory T cell responses.^[13]^ Specific miRNA expression profiles (over-expressed miR-17-5p and miR-193a; lower expression of miR-497, miR-1, and miR-126) in CD4+, CD8+, and B cells have been noted to regulate the immune-pathogenesis mechanism of MS.^[14]^ Increased miR-128, miR-27b, and miR-340 expressions in naïve or memory CD4(+) T cells can inhibit the development of Th2 cells and promote proinflammatory Th1 responses.^[15]^ miR-20b was found to reduce Th17 differentiation, resulting in the modulation of RORγt and STAT3 pathways. Furthermore, studies on RRMS noted upregulated expressions of key miRNA bio-genesis molecules DGCR8, Dicer and Drosha, further strengthening the central function of miRNAs in MS pathophysiology.^[16]^ Various TGFβ-targeting miRNAs in naïve CD4 T cells appear to slow the development of regulatory T cells, thereby increasing the risk of MS.^[17]^ Interestingly, varying miR-21 and let-7 expressions were detected in different phases of MS.^[18,19]^ All the findings above presented that miRNA can regulate the development and differentiation of immune cells modulating immune system responses.^[20]^ These results indicated that miRNAs play key roles in MS biology and remain potential therapeutic candidates.^[21]^

The development of new drugs is a challenging and lengthy processes that often encounters high failure rates and costs. Therefore, studies on drug repurposing have garnered significant attention.^[22,23]^ One such example of successful drug repositioning is sildenafil which was first intended to be used as an antihypertensive. Sildenafil^[24]^ is currently sold under the trade name of Viagra, a poster-child drug of erectile dysfunction as well as pulmonary arterial hypertension.^[25]^ A key prerequisite for drug repurposing efforts is to first identify potential links between drugs and targets. Peng et al^[26]^ pioneered the Predicting Drug-Target Interactions using Multi-Information Fusion. Hu and Agarwal^[27]^ designed a large-scale disease-drug network for efficient and effective drug repositioning as well as drug target/pathway identification using GEO datasets. Cao et al^[28]^ constructed a miRNA-regulated drug-pathway network to reveal potential pre-existing medications that may be used to treat myasthenia gravis. Liu et al^[29]^ constructed a drug network based on the indications enriched by network neighbors for drug repositioning using side effects. Dopaminergic agents have also been repurposed for MS.^[30]^ However, few studies have focused on miRNA-regulated drugpathway networks to explore potential candidates which may be repurposed for the intention of treating MS.

The current investigation identifies risk genes or risk miRNA-target genes which are known to be associated with RRMSrelated pathways. Potential drug candidates were identified based on the RRMS risk genes. This was followed by the construction of a miRNA-regulated drug-pathway network through genes (MDNG). The MDNG was then analyzed using the Z-values between MS and the drugs, which resulted in a number of likely MS drug candidates. Lastly, these candidate drugs were further investigated with regards to their likely mechanisms of action in MS treatment. Our findings provide a fresh perspective in the realm of MS therapy.

## 2. Materials and methods

The design of the research is presented in Figure 1.

**Figure:**
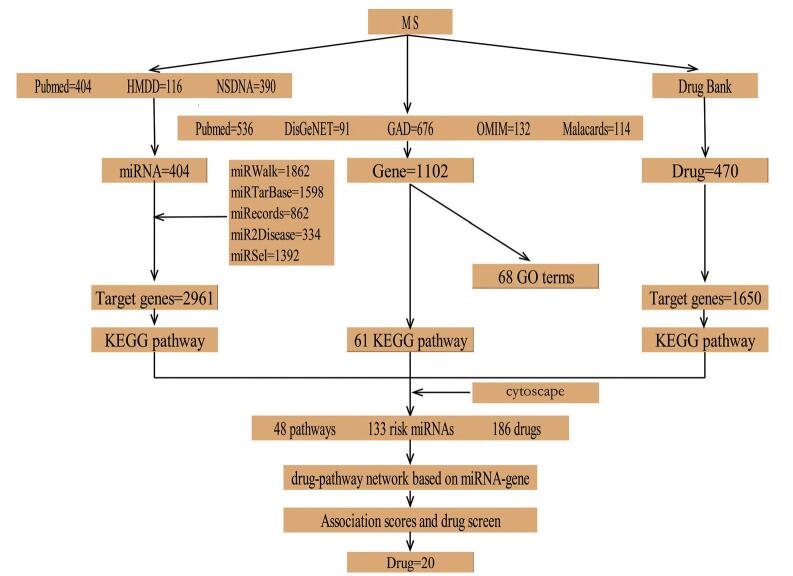
**Figure 1.** The routes and block diagram of the research.

### 2.1. MS risk gene collection

The MS risk genes were obtained from browsing the PubMed database. The terms RRMS as MeSH terms and English as language and “homo sapiens” were used to search the literature in Pubmed. A total of 59,321 items were obtained from Pubmed prior to September 1, 2020. We selected genes which were identified with the use of reliable experimental methods (such as microarrays, enzyme linked immunosrbent assay, Real Time - Polymerase Chain Reaction, or Western Blot) and were documented in at least 5 RRMS samples. Meanwhile, we also compiled various risk genes by searching databases such as DisGeNET (http://www.disgenet.org), Genetic Association Database (http://geneticassociationdb. nih.gov), Online Mendelian Inheritance in Man (https://omim.org), andMalacards (https://www.malacards.org). These methods yielded a total of 1102 MS risk genes.

### 2.2. MS risk miRNAs and miRNA targets

MS risk miRNAs were also acquired via 2 separate methods. Literature published prior to September 1, 2020 were manually extracted using the keywords “miRNA” and “multiples sclerosis” or “microRNA” and “multiples sclerosis” or “miR” and “multiples sclerosis” in PubMed (https://www.ncbi.nlm.nih.gov/pubmed). Furthermore, MS risk miRNAs were extracted from the Human microRNA Disease Database (HMDD v3.0) (http://www.cuilab.cn/hmdd) and the Nervous System Disease database (NSDNA, http://www.bio-bigdata.net/nsdna/).^[31]^ All miRNAs have been identified to be associated with active or progressive MS. Human miRNA target data were obtained from experimentally validated miRNA target databases, including miRWalk,^[32]^ miRTarBase,^[33]^ miRecords,^[34]^ miR2Disease,^[35]^ and miRSel.^[36]^

### 2.3. Human drug target data

The DrugBank database (version 5.1.1, https://www.drugbank.ca/) was explored for information regarding all drugs and their target genes. Drug target results were specified to include only the species “Homo sapiens” and only included drugs with more than 5 target genes.

### 2.4. MS pathway analysis

The Database for Annotation, Visualization, and Integrated Discovery allowed for the identification of MS risk pathways through functional enrichment of MS gene lists. The Kyoto Encyclopedia of Genes and Genomes (KEGG) pathway enrichment of MS risk genes were determined based on a *P* value cutoff of .05. AmiGO2 (http://amigo.geneontology.org/) was used to carry out gene ontology (GO) biological process enrichment was performed using. An false discovery rate (FDR)-value cut-off of <0.05 was used to indicate significant GO enrichment. We then constructed a network consisting of MS risk genes, miRNAs, and drugs target genes pathways using Cytoscape 3.5.1. The relevant network characteristics were analyzed with the Network Analysis plugin.

### 2.5. Association scores and drug screen

The MDNG network was composed of MS risk genes pathways, miRNA-genes pathways and drug-genes pathways. Based on this network, the drugs’ association scores were calculated by the following formula-1:



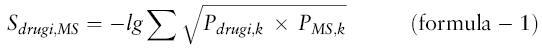



In the formula, *P_MS,K_* is interpreted as the *P* value of MS enriched in pathway “k”. *P_drugi,k_* is the enriched *P* value of drug “I” on pathway “k”, and the “k” is presented as the pathway most significantly impacted by both MS risk genes and drug “i” target genes. This formula was used to obtain the S value of each candidate drug.

Furthermore, the Z-scores of the drug candidates, MS, as well as random pathway permutations were used to determine the specificity of the aforementioned association. Furthermore, 10,000 random pathway rankings were performed to generate random pathway drug profiles. The *S_random,drugi_* for each profile was calculated according to formula (1). The Z score was calculated by the following formula-2:



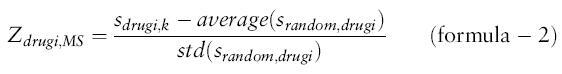



The specificity of the association between MS and drug candidate was determined via the Z value. In the formula above (2), the average (*S_random,drugi_*) represents the average association score between drug “i” and random cases, while *std*(*S_random,drugi_*) is the standard variation of association between drug “i” and random cases. A more significant association between MS and the drug is denoted by a higher *Z*-score. The Z-value >1.96 (*P* < .05) was regarded as the cutoff for drug screening.

### 2.6. Statistical analysis

KEGG pathway enrichment was performed using of *P* value cutoff of <.05. Significant GO enrichment was performed using a threshold of FDR-value <0.05. Cytoscape 3.5.1 was used to visualize the network. The relevant network characteristics were analyzed with the Network Analysis plugin. S value of each candidate drug and Z score were calculated by RStudio. *P* < .05 was considered statistically significant.

## 3. Results

### *3.1. A comprehensive profile of MS risk genes, miRNAs,* *and pathways*

One thousand one hundred two MS risk genes were obtained using the public information network. All the genes were confirmed by established experiment protocols to have an association with MS. Of these, 536 risk genes were manually extracted from the PubMed database. Other risk genes were obtained from public databases (DisGeNET = 91, Genetic Association Database = 676, Online Mendelian Inheritance in Man = 132, Malacards = 114). KEGG enrichment analysis of these genes revealed 61 MS risk pathways. Figure 2A demonstrates the top 15 pathways and their *P* values. Figure 2B depicts the 4 major categories that the pathways fell into, which were “human diseases”, “Organismal Systems”, “Environmental Information Processing”, and “Metabolism”. A majority of these pathways were related to “Human Diseases: Immune diseases”, “Human Diseases: Cancers”, “Human Diseases: Infectious diseases”, and “Organismal Systems: Immune system”. Among them, hsa05330 (allograft rejection) was the primary significantly enriched pathway, which involved a total of 31 MS risk genes (Fig. 2C).

**Figure:**
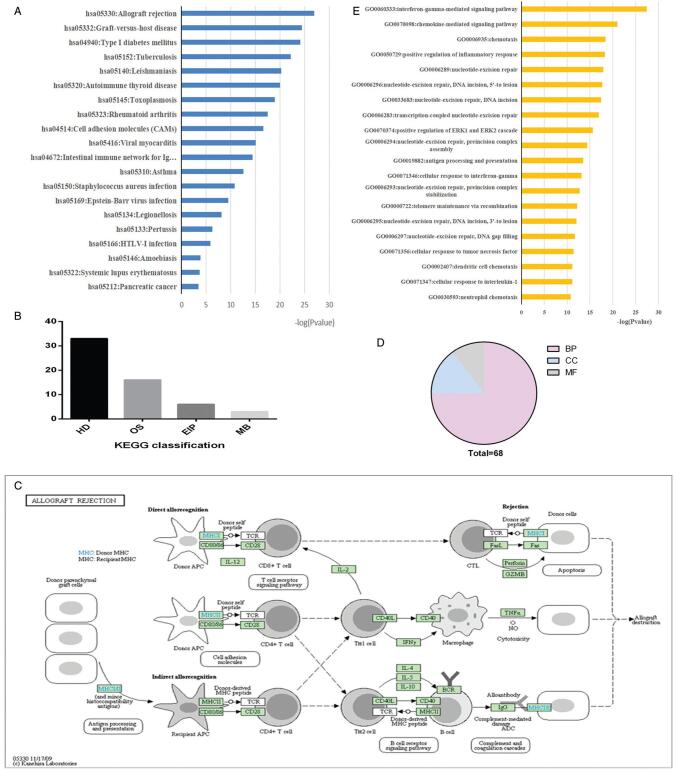
**Figure 2.** Multiple sclerosis (MS) risk pathways. (A) Top 15 KEGG pathways enriched by MS risk genes (*P* < .05). (B) Classification of MS risk pathways. (C) Dissection of the MS risk pathways (hsa05330: allograft rejection). (D) Classification of MS GO terms. (E) The top 15 of biological processes. BP = biological processes, CC = cellular components, EIP = Environmental Information Processing, GO = gene ontology, HD = human diseases, KEGG = Kyoto Encyclopedia of Genes and Genomes, MB = metabolism, MF = molecular functions, OS = organismal systems.

These risk genes were then subjected to a functional enrichment analysis. We uncovered a total of 68 GO terms (Fig. 2D) (FDR < 0.05), which encompassed 51 biological processes (BP), 7 molecular functions, and 10 cellular components. The top 15 of BP were presented in Figure 2E.

Most genes are enriched in vital BP, such as positive regulation of inflammatory response, chemotaxis, chemokine-mediated signaling pathway, interferon-gamma-mediated signaling pathway, and nucleotide-excision repair. The results reveal that BP in MS are mainly related to immunity, inflammation, and DNA damage.

After eliminating the duplicate data, 404 MS risk miRNAs were obtained (Pubmed = 404, Human microRNA Disease Database = 115, NSDNA = 390). Three hundred eighty-five MS risk miRNAs were found to have target genes. Considering the massive data sample, we set up the screening conditions. MiRNAs with more than 5 target genes were retained. We identified miRNA-regulated risk pathways using all target genes. Based on these genes, 162 MS risk pathways were identified using KEGG enrichment analysis. Among them were 138 miRNAs and their 2923 experimentally validated target genes. Furthermore, we constructed 12,101 miRNA-gene interaction pairs. We then constructed a miRNA-pathway regulating network for MS. The miRNA which regulated the highest number of risk pathways was miRNA-155-5p. There were 262 experimentally validated target genes of miR-155-5p. Among them, 63 genes were associated with MS. The top pathway identified was has04660: T cell receptor signaling pathway. Twenty-seven target genes were enriched in hsa04660: T cell receptor signaling pathway (IL4, PIK3CG, SOCS6, HRAS, IL5, TNF, PIK3CB, PIK3CD, CTLA4, NFKBIA, NFKB1, CDK4, IL10, AKT1, FOS, MAPK1, IL17, JUN, MAP3K8, MAPK3, IFNG, RHOA, ZAP70, PIK3CA, CD4, PIK3R1, IL2). The 27 genes were analyzed to construct the protein-protein interactions network via STRING interactome (https://string-db.org/). The visualized network is depicted in Figure 3. Using the Cytoscape plug-in MCODE, 2 sub-network modules were extracted from the protein-protein interaction network. Eighteen nodes and 134 edges were included in module A (score = 15.756). The nodes with degree greater than 20 included AKT1 (degree = 25), CD4 (degree = 22), IL-4 (degree = 22), JUN (degree = 22), HARS (degree = 22), TNF (degree = 21). Module B contained 6 nodes and 11 edges, including PIK3R1 (degree = 22). Most of the genes above were associated with autoimmune reactions. For instance, the expression of AKT-1 and phosphorylated-Akt1 (p-Akt1) decreased in CD4+ T cell and regulatory T (Treg) cells of RRMS.^[37]^ Numerous studies have found IL-4 and IL-4R to be associated with MS.^[38]^ Increased TNF levels were previously the meninges and CSF in MS.^[39]^ Moreover, miR-155-5p is known to regulate the pathological process of MS by targeting the aforementioned genes. Sanders et al^[40]^ reported miR-155-5p downregulation in SPMS CD4+ T cells, which targeted SOCS6. Decreased immune system activation seen in progressive MS may be the result of raised SOCS6 expressions in SPMS CD4+ T cells. Ksiazek-Winiarek et al^[41]^ proposed that IL-17 exerts protective activity by downregulating miR-155-5p in an EAE model. Due to the central role of CD4+ T cells in MS,^[42]^ these results highlighted the importance of miR-155-5p in MS. For the above-mentioned reasons, miR-155-5p may function as a potential MS biomarker.

**Figure:**
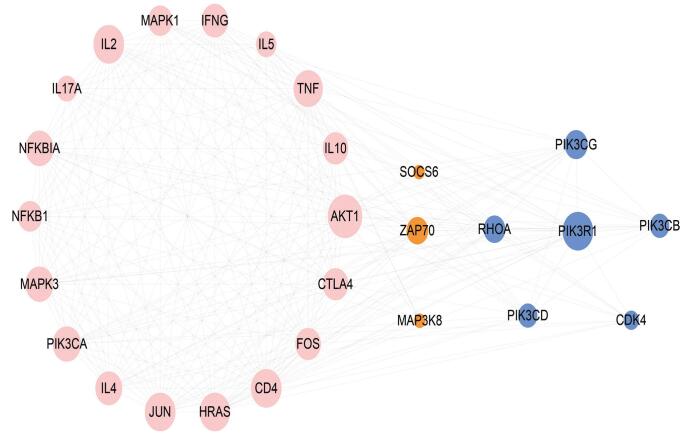
**Figure 3.** Two sub-modules extracted from protein-protein interaction (PPI) network. The pink nodes were enriched in module A, the blue nodes were enriched in module B. The size of the nodes corresponded to degree value, larger node has higher degree value.

### 3.2. Topological features of the MDNG

Four hundred seventy drugs, 1650 drug targets, and 6165 drugtarget pairs were obtained from Drug Bank. Database for Annotation, Visualization, and Integrated Discovery was then used to determine drug pathways of statistical significance based on its target genes. When the drug-genes pathways overlapped with those that were enriched by the MS risk genes and miRNA targets, these drugs were selected for follow-up experiments. A total of 186 drugs with common pathways between MS risk genes and miRNA target genes were noted. An MDNG was constructed based on these drug-pathway and miRNA-pathway associations (Fig. 4A) and comprised of 367 nodes and 2833 interactions, which included 48 pathways, 133 MS risk miRNAs, and 186 drugs.

**Figure:**
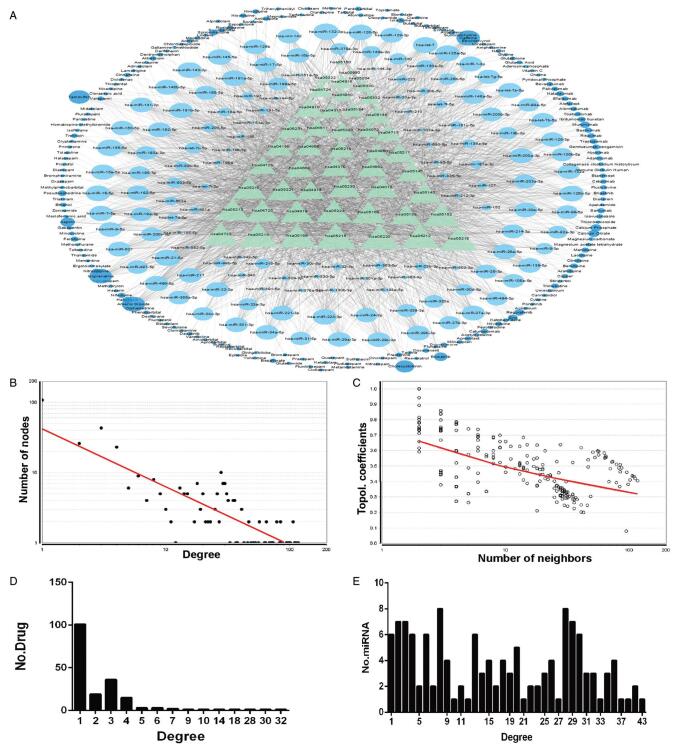
**Figure 4.** MDNG in MS. (A) miRNA-regulated drug-pathway network through genes (MDNG) in multiple sclerosis (MS). Network was consisted of miRNAs, drugs, and pathways. Wathet circle represents miRNAs, mazarine circle represents drugs, and green triangles represents pathways. (B) Degree of distribution for all nodes in the MDNG. (C) Topological coefficients for all nodes in the MDNG. (D) Degree distribution of the drug nodes. (E) Degree distribution of the miRNA nodes.

The topological structure of the network was then subjected to further scrutiny including the degree of distribution and topological coefficient of the network. The degree distribution was in concurrence with the law of distribution [f(x) = 41.042x^[-0.823]^] based on all MDNG nodes (Fig. 4B).

Meanwhile, the topological coefficient was calculated to measure the betweenness of the inter-nodal network links (Fig. 4C). There appeared to be a decreasing topological coefficient with an increasing number of neighboring nodes. Further analysis on the degree of drug distribution revealed that majority of drugs (171/186, 91.93%) were connected by only a few pathways. The degree of distribution of the miRNAs is depicted in Figure 4E. There were a number of miRNAs that were associated only with a few number of drugs, indicating that these molecules may regulate multiple drug-associated pathways. We conclude that these findings may be represented by drug resistance or sensitivity.

### *3.3. miRNAs and drugs regulate key MS pathways via* *genes*

In MDNG, the pathway (hsa05215: Prostate cancer) possessed high connectivity and was regulated by miRNAs and 10 drugs through target genes pathways. There were 10 MS risk genes involved in the hsa05215 pathway, including MAPK1, AR, TCF7, CASP9, BCL2, TP53, FOXO1, TCF7L2, AKT3, and PIK3R1 (Fig. 5). This pathway was explored in detail with the key genes co-regulated by MS risk miRNAs and drugs were examined. Several MS risk genes were targeted by drugs and miRNAs, suggesting that MS risk pathways may be modulated by specific gene-modifying drugs and miRNAs. A total of 3 MS risk genes (PIK3R1, MAPK1, TP53) were found to be regulated by multiple miRNAs and drugs. In addition, 59 MS risk genes regulated the hsa05152: Tuberculosis pathway, and 24 drugs and 75 miRNAs participated in this pathway (Fig. 6). The result may indicate that hsa05215: Prostate cancer and hsa05152: Tuberculosis play core roles in the network and suggest the important roles that these pathways may have in MS pathogenesis.

**Figure:**
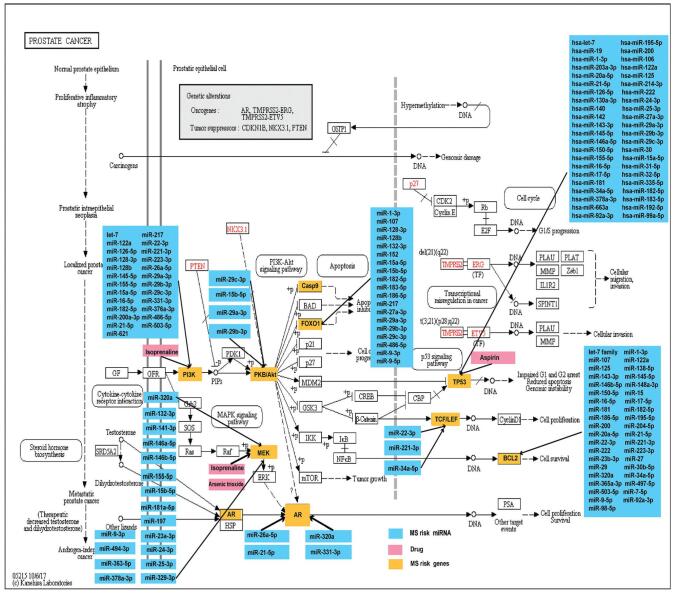
**Figure 5.** Identifying multiple sclerosis (MS) risk genes regulated by drugs or miRNAs in the hsa05215 pathway. Yellow represents the MS risk genes regulated by MS risk miRNAs or drugs. Blue represents the MS risk miRNAs associated with MS risk genes. Pink represents the drugs associated with MS risk genes.

**Figure:**
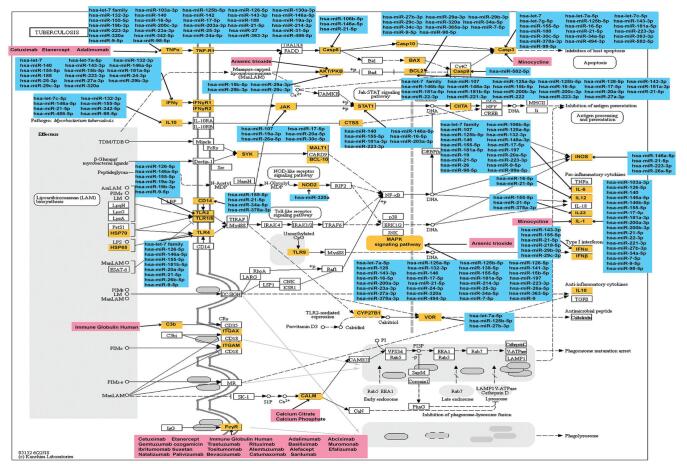
**Figure 6.** Identifying multiple sclerosis (MS) risk genes regulated by drugs or miRNAs in the hsa05152 pathway. Yellow represents the MS risk genes regulated by MS risk miRNAs or drugs. Blue represents the MS risk miRNAs associated with MS risk genes. Pink represents the drugs associated with MS risk genes.

### 3.4. Identification of drug repurposing candidates for MS

According to the MDNG, we determined a drug repurposing strategy and identified 20 drug repurposing candidates for MS with Z scores >1.96 (Table 1). We confirmed the reliability of drug candidates using 2 methods: searching the ClinicalTrials. gov database (https://www.clinicaltrials.gov/) or consulting the relevant literature in PubMed. Among the candidates, 4 drug candidates (human immunoglobulin, aspirin, alemtuzumab, minocycline) have been studied in MS clinical studies. Intravenous immunoglobulin is used for the treatment of immune deficiencies, as well as autoimmune and inflammatory disorders. In the clinical setting, intravenous immunoglobulin has been confirmed to be effective in treating MS.^[43]^ There are 33 clinical trials studying the treatment effects of MS in ClinicalTrials.gov. Aspirin is a cyclooxygenase inhibitor that has antipyretic and anti-inflammatory properties by suppressing prostaglandin production. There were 4 clinical trials studying the efficacy and safety of aspirin in MS. Aspirin has been hypothesized to be a useful adjuvant in ameliorating disease progression in MS.^[44,45]^ Alemtuzumab is a humanized monoclonal antibody used in B-cell chronic lymphocytic leukemia treatment. Both US and European Union organizations have authorized alemtuzumab for the treatment of MS. In clinical trials, it has been confirmed that alemtuzumab is an appropriate treatment choice for RRMS.^[46]^ Furthermore, certain trials have found minocycline to be able to reduce the risk of conversion from clinically isolated syndrome to MS.^[47]^ In addition, copper and caffeine were eliminated. Finally, 14 drugs were identified and presented in Table 1. Other drug candidates were noted to be related to the treatment of autoimmune diseases or inflammatory diseases. For instance, etanercept is known to inhibit TNF-alpha-induced pro-inflammatory activity in rheumatoid arthritis^[48]^ patients and reduce the severity of psoriasis.^[49]^ Adalimumab is a novel TNF-alpha antibody that can slow the progression of rheumatoid arthritis^[50]^ and alleviate the severity of ankylosing spondylitis. Tositumomab binds to the CD20 antigen and leads to selective killing of Bcells.^[51]^ These results lend credibility to our findings.

**Table 1:** The candidate drugs.

**Drug candidates**	**Accession number**	**Description**	**Z-score**
Etanercept	DB00005	Etanercept is indicated for the treatment of moderately to severely active rheumatoid arthritis in adults, chronic moderate to severe plaque psoriasis in adults or ankylosing spondylitis.	15.83378594
Abciximab	DB00054	Abciximab binds to the glycoprotein IIb/IIIa receptor of human platelets and inhibits platelet aggregation by preventing the binding of fibrinogen, von willebrand factor, and other adhesive molecules.	13.91999357
IVIG	DB00028	In the clinical setting, intravenous immunoglobulin has been confirmed to be effective in the treatment of MS.	10.19770137
Catumaxomab	DB06607	Catumaxumab is a trifunctional monoclonal antibody developed for use in cancer treatment. It was initially authorized for the treatment of malignant ascites.	10.18068379
Adalimumab	DB00051	Adalimumab is a subcutaneously administered biological disease modifier for the treatment of rheumatoid arthritis and other chronic debilitating diseases mediated by tumor necrosis factor.	9.102887099
Alefacept	DB00092	Alefacept can be used for treatment of moderate to severe chronic plaque psoriasis.	7.628269542
Aspirin	DB00945	Aspirin has been hypothesized to be a useful adjuvant in ameliorating disease progression.	6.534323191
Palivizumab	DB00110	Palivizumab was used for prophylaxis of respiratory diseases caused by respiratory syncytial virus.	6.21635952
Alemtuzumab	DB00087	In clinical trials, it has been confirmed that alemtuzumab is an appropriate treatment choice in RRMS.	6.162288965
Sarilumab	DB11767	Sarilumab is indicated for the treatment of moderate to severe rheumatoid arthritis in combination with methotrexate.	5.74643132
Bevacizumab	DB00112	Bevacizumab is a recombinant humanized monoclonal IgG1 antibody that binds to and inhibits the biologic activity of human vascular endothelial growth factor.	5.479469509
Efalizumab	DB00095	Efalizumab is indicated for the treatment of adult patients with moderate to severe chronic plaque psoriasis.	5.447839061
Pseudoephedrine	DB00852	It has been used in the treatment of several disorders including asthma, heart failure, rhinitis, and urinary incontinence.	5.269473318
Tositumomab	DB00081	Tositumomab is indicated for treatment of non-Hodgkin’s lymphoma.	4.336066113
Minocycline	DB01017	Certain trials have found minocycline to be able to reduce the risk of conversion from clinically isolated syndrome to MS.	4.079792945
Thalidomide	DB01041	Thalidomide originally introduced as a non-barbiturate hypnotic, but withdrawn from the market due to teratogenic effects. At present, it was used for a number of immunological and inflammatory disorders.	2.806803806
Muromonab	DB00075	For treatment of organ transplant recipients, prevention of organ rejection.	2.708730123
Isoprenaline	DB01064	It is used mainly as bronchodilator and heart stimulant.	2.576112147
IVIG = intravenous immunoglobulin; MS = multiple sclerosis, RRMS = remission type multiple sclerosis.			

### *3.5. Mechanism dissection of drug candidates and* *miRNAs in MS*

We constructed a network comprising of drug, drug target genes, MS risk miRNA, miRNA target gene, and MS risk pathways as a convenient means to investigate the complex relationships and underlying biological pathways that exist between miRNAs, genes, and drugs. We further designed a layered network between alemtuzumab and MS to determine the potential treatment efficacy of alemtuzumab in MS. The network demonstrated that the treatment effects of alemtuzumab are mainly involved in 3 pathways (hsa05140: Leishmaniasis, hsa05133: Pertussis, hsa05152: Tuberculosis). Abciximab, alefacept, palivizumab, bevacizumab, efalizumab, and tositumomab were found to share the same pathways with alemtuzumab. Therefore, we only presented the alemtuzumab pathway (Fig. 7). These findings suggest that these drugs may be efficacious if administered in combination given the involvement of similar pathways. Seventy MS risk genes were found to be related to the 3 pathways. Thirtyseven risk genes regulated by MS risk miRNAs were noted, and this included BCL2, BAX, CASP3, IL1A, IL1B, IL4, IL6, IFNA1, IFNB1, IFNG, JAK1, JAK2, MAPK1, STAT1, TNF, TGFB1, TGFB2, TLR2, TLR4, and PTGS2. BCL2 encodes an important outer mitochondrial membrane protein responsible for inhibiting apoptotic cell death in some cells such as lymphocytes. BCL2 has been implicated in several autoimmune conditions including autoimmune thyroid diseases^[52]^ and primary biliary cholangitis.^[53]^ BCL2 was regulated by 58 MS risk miRNAs, such as let-7 family, hsa-miR-146, and hsa-miR-15/miR-16. Another vital immune system signal molecule is TNF, which is a potent proinflammatory cytokine belonging to the tumor necrosis factor superfamily. The TNFRSF1A/TNFR1 and TNFRSF1B/TNFBR receptors are known to bind to members of this superfamily. TNF is an important drug target for autoimmune and immunemediated inflammatory diseases.^[54]^ Fourty-six MS risk miRNAs were found to target TNF expression. Furthermore, the protein encoded by casp3 is a cysteine-aspartic acid protease and is processed by caspases 8, 9, and 10. Casp3 was regulated by 26 MS risk miRNAs, including let-7 family, hsa-miR-155-5p, and hsa-miR-21-5p. These results suggest that miRNAs participate in MS risk pathway through target genes. Similarly, we constructed a layered network that included minocycline (Figure S1, Supplemental Digital Content, http://links.lww.com/MD/G660), etanercept (Figure S2, Supplemental Digital Content, http://links.lww.com/MD/G661), catumaxomab (Figure S3, Supplemental Digital Content, http://links.lww.com/MD/G662), sarilumab, and MS. Catumaxomab and sarilumab were found to share the same pathways (hsa05140; hsa05322; hsa05152; hsa05150). Meanwhile, we found that almost all drugs are related to hsa05152: Tuberculosis. The results again demonstrated that hsa05152 is a critical mediator in MS pathophysiology.

**Figure:**
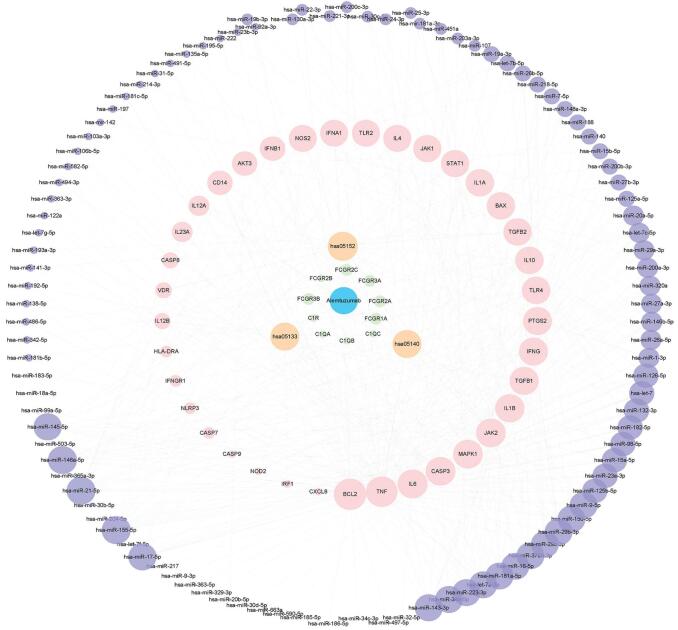
**Figure 7.** Layered network model linked slemtuzumab with multiple sclerosis (MS). Abciximab, alefacept, palivizumab, bevacizumab, efalizumab, and tositumomab share the same pathways with alemtuzumab. The bigger circle means the higher weights and plays a more important role in the network. Orange represents the pathway, blue represents the drug, green represents the drug target gene, pink represents MS risk genes regulated by MS miRNAs, purple represents MS risk miRNAs.

## 4. Discussion

MS is a complicated autoimmune disease that demonstrates a varied constellation of symptoms.^[1]^ Because of the high disability rates of MS, it is necessary to identify the safe and effective medicines for the treatment of MS. MS has no cure, with current strategies seeking to slowing down disease progression and reducing relapse rates.^[6]^ Glucocorticoid, plasma exchange, and immunoglobulin are currently used to manage acute symptoms of MS. DMTs such as teriflunomide,^[55]^ injectable recombinant interferon beta-1b,^[56]^ or alemtuzumab^[46]^ are recommended for MS in remission. However, these treatment modalities are costly and harbor several adverse effects. Additionally, novel drug innovations are marred by lengthy development periods, along with costs and failure rates expensive and can induce side-effects. Meanwhile, RRMS patients will suffer from recurrent attacks. Finally, they have poor quality of life and worse prognosis. Therefore, pursuing drug repurposing and repositioning for RRMS is preferential.

Among KEGG pathway enriched by MS risk genes, hsa05330 (related to allograft rejection) was the main significantly enriched pathway. Allograft rejection can be activated by the direct pathway or indirect pathway.^[57]^ The direct pathway would activate host CD4 or CD8 T cells (predominantly CD4 T cells). CD4 T cells or CD8 T cells are activated by reacting with MHC molecules.^[58]^ T cells can contribute to graft rejection by activating macrophages, which cause tissue injury and fibrosis.^[59]^ Similarly, CD+4 or CD+8 T cells are important in the development of MS.^[60,61]^ Immune cells have destructive effects on CNS myelin and oligodendrocytes.^[62]^ Therefore, it is unsurprising that a majority of MS risk genes are enriched in hsa05330 (allograft rejection).

This study represents a comprehensive catalog of MS risk genes and miRNAs. MS risk pathways were then derived from these genes and miRNA-target genes. MiR-155-5p as immunoregulatory microRNA was observed downregulated in SPMS or EAE model. Singh et al^[63]^ observed that miR-155-5p was increased before the early stage of MS in the EAE model and declined gradually in the pathological process of EAE. In addition, the downregulating of miR-155-5p targeting with IL17 can be confirmed in EAE.^[41]^ Decreased immune system activation and downregulation of miR-155-5p in progressive MS may be the result of raised SOCS6 expressions in SPMS CD4+ T cells.^[40]^ These results highlighted the importance of miR-155-5p in MS.

A large proportion of these pathways are associated with cancer, inflammation, and immune disease. Drugs possessing shared pathways between miRNA target genes and MS risk genes were then identified by constructing MDNG. Further key MS pathways were then discerned based on the topological features of the MDNG. miRNAs and drugs which synergistically modulated MS risk pathways were then analyzed. The hsa05215: Prostate cancer pathway was noted to have high connectivity. MS risk genes (MAPK1, AR, TCF7, CASP9, BCL2, TP53, FOXO1, TCF7L2, AKT3, and PIK3R1) were also involved. PIK3R1, MAPK1, and TP53 were found to be regulated by multiple miRNAs and drugs. PIK3R1 is involved in the PI3Kδ pathway, and is involved in regulating multiple cellular processes, including growth, metabolism, differentiation, proliferation, and so on. The PI3K pathway defects are known to result in immunodeficiency and immune dysregulation.^[64]^ Overactive MAPK activity induces microglial malfunction and leads to locoregional demyelination and is a significant contributor to MS pathophysiology.^[65]^ Fifty-nine MS risk genes participated in the hsa05152: Tuberculosis pathway, and were primarily the HLA-II, toll-like receptors, caspase family, BCL2 family, interleukin family, interferon family, and so on. Some proinflammatory cytokines have been confirmed in the development of MS, such as IL-1, IL-6, IL-12, and TNF.^[66]^ Toll-like receptors recognized and presented antigens to initiate an inflammatory response.^[67]^ The results suggest that the hsa05215: Prostate cancer and hsa05152: Tuberculosis play core roles in the network and indicate that the pathways are extremely important in the pathogenesis of MS.

Additionally, we constructed a drug repurposing strategy and identified 20 repurposed drug candidates for MS. Most of the drugs are related to immune diseases, inflammatory, or CNS disease. Nearly all the drugs we screened participated in the hsa05140: Leishmaniasis pathway. The drugs were also found to play regulatory roles on genes involved in the hsa05140: Leishmaniasis pathway. Activation of the toll-like signaling pathway, MAPK signaling pathway, or Jak-STAT signaling pathway were found to lead to macrophage deactivation, antigen presentation failure, impaired microbial killing, and prevention of the Th1 immune response (Fig. 8). Other drugs were shown to effective MS treatment modalities as evidenced by clinical trials or related literature.^[68]^ Alemtuzumab as CD52 antibody that induces the depletion of T and B cell and not attack monocytes, macrophages, and so on. Alemtuzumab can produce a marked effect by acting on C1 complex to inhibit the opsonization and phagocytosis in hsa05133: Pertussis. Then the drug will improve therapeutic effect in the long term.^[69]^ Alemtuzumab is a humanized monoclonal antibody that is also indicated in treating MS.^[70]^ In a cohort study, researchers evaluated the therapeutic effectiveness of 4 drugs. Therapeutic efficacy of alemtuzumab and natalizumab were found to be superior to first line therapy of interferon beta-1a or fingolimod.^[71]^ These results again provided evidence that the drugs we screened were accurate and reliable. Given the complexity of this disease, it is therefore important to adopt a multi-prong approach in treating MS that is not limited to immunotherapy. Feinstein et al^[7]^ presented that concurrent and synergistic treatments are needed in MS. This hypothesis is consistent with the complexity of RRMS risk pathways and the diversity of drugs that were involved in this study. It would be beneficial for future studies to investigate the exact utility of our proposed drug candidates in RRMS.

**Figure:**
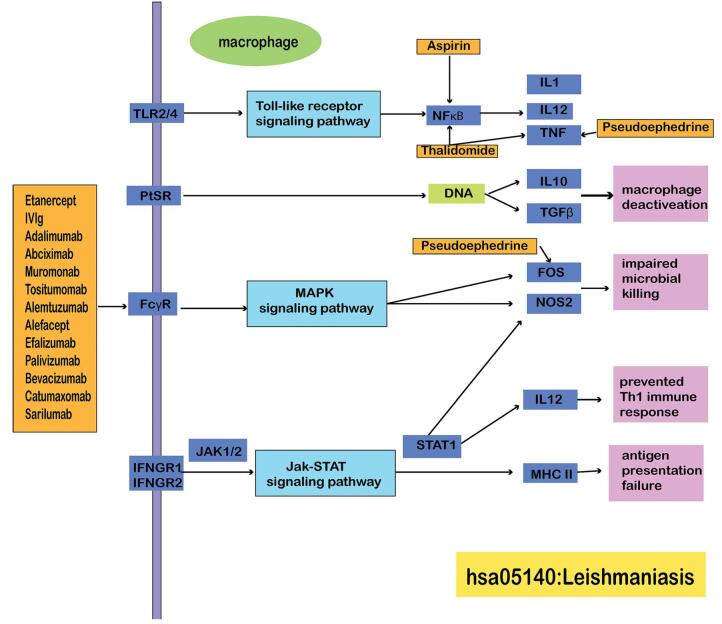
**Figure 8.** Drugs play roles in hsa05140 pathway through genes.

## 5. Limitations

The main limitation of our study is that we have not verified the effectiveness and safety of drugs in EAE models. The databases used may also be incomplete, further limiting our study. Therefore, future studies should be aimed at verifying the effectiveness of screening drugs in autoimmune encephalomyelitis models. Finally, more studies are required to validate the clinical application of these identified drug candidates.

## 6. Conclusions

This investigation presents a comprehensive collection of MS risk genes and miRNAs. Based on these genes, several MS risk pathways were delineated to aid in identification of novel MS treatment strategies. An MDNG was constructed to better understand the relationship among genes, miRNAs, pathways, or drugs and to determine existing drugs that may be repurposed into MS therapeutic agents. We provide a refreshing outlook on MS that has the potential to unlock several promising new MS therapies.

## Author contributions

**Conceptualization:** Xiaotong Kong, Jianjian Wang, Yuze Cao, Ming Bai, Shuang Li, Lihua Wang.

**Data curation:** Xiaotong Kong, Jianjian Wang, Yuze Cao, Xiaoyu Lu, Chunrui Bo, Ming Bai, Shuang Li, Yang Jiao, Lihua Wang.

**Formal analysis:** Xiaotong Kong, Jianjian Wang, Xiaoyu Lu, Chunrui Bo, Yang Jiao.

**Funding acquisition:** Jianjian Wang, Lihua Wang.

**Investigation:** Xiaotong Kong.

**Methodology:** Xiaotong Kong, Huixue Zhang, Yang Jiao.

**Project administration:** Xiaotong Kong.

**Resources:** Xiaotong Kong, XiaoMing Zhang.

**Software:** Xiaotong Kong, Jianjian Wang, Yuze Cao, Xiaoyu Lu, Huixue Zhang, XiaoMing Zhang, Ming Bai, Shuang Li.

**Supervision:** Jianjian Wang, Chunrui Bo, Ming Bai, Shuang Li.

**Validation:** Xiaotong Kong, Jianjian Wang, XiaoMing Zhang, Chunrui Bo, Ming Bai, Shuang Li.

**Visualization:** Xiaotong Kong, Jianjian Wang, Yuze Cao, Huixue Zhang, XiaoMing Zhang, Chunrui Bo, Shuang Li.

**Writing - original draft:** Xiaotong Kong, Jianjian Wang, Yuze Cao, Huixue Zhang, Chunrui Bo.

**Writing - review & editing:** Xiaotong Kong, Jianjian Wang, Yuze Cao, Chunrui Bo.
